# A Rare Presentation of Adult Primary Leptomeningeal Medulloblastoma: Case Report

**DOI:** 10.1155/crip/8937543

**Published:** 2025-09-08

**Authors:** Grace E. Hey, Megan E. H. Still, Rachel S. F. Moor, Amanda N. Stanton, Duane A. Mitchell, Brent A. Orr, Jesse L. Kresak, Anthony A. Yachnis, Tara Massini, Ashley P. Ghiaseddin

**Affiliations:** ^1^University of Florida College of Medicine, Gainesville, Florida, USA; ^2^Lillian S. Wells Department of Neurosurgery, University of Florida College of Medicine, Gainesville, Florida, USA; ^3^Lillian S. Wells Department of Neurosurgery, Preston A. Wells Jr. Center for Brain Tumor Therapy, UF Clinical and Translational Science Institute, University of Florida, Gainesville, Florida, USA; ^4^Department of Pathology, St. Jude Children's Research Hospital, Memphis, Tennessee, USA; ^5^Department of Radiology, University of Florida College of Medicine, Gainesville, Florida, USA; ^6^Department of Pathology, Immunology, and Laboratory Medicine, University of Florida, Gainesville, Florida, USA

**Keywords:** leptomeningeal medulloblastoma, medulloblastoma, neuro-oncology, neurosurgery

## Abstract

Medulloblastomas are tumors of the posterior fossa that have a propensity to develop leptomeningeal metastases along the spinal cord, commonly known as “drop metastases.” Medulloblastoma accounts for approximately 1%–2% of all adult brain tumors, and reports of primary leptomeningeal medulloblastoma are extremely limited. Herein, we present a rare case of a 34-year-old woman diagnosed with multifocal primary spinal leptomeningeal medulloblastoma without cranial involvement.

## 1. Introduction

Medulloblastoma is a heterogeneous group of primary malignant brain tumors that originate in the cerebellum or posterior fossa [1]. The exact etiology and pathophysiology of medulloblastoma are poorly understood but are believed to involve genetic mutations and signaling pathway disruptions, particularly in the wingless-related integration, sonic hedgehog (SHH), and Group 3 and Group 4 pathways [2]. Medulloblastoma predominantly occurs in children but represents 1%–2% of all primary adult brain tumors [3]. The overall prognosis of medulloblastoma in adults is generally favorable, with a 10-year survival rate of approximately 70% [3–5]. Treatment regimens typically involve a multimodal approach, combining surgery, radiation therapy, and chemotherapy [3].

Medulloblastomas are known to develop leptomeningeal metastases, also known as “drop metastases”, which refer to the dissemination of tumor cells through the cerebrospinal fluid to distant sites within the central nervous system, particularly along the spinal cord [6–8]. These metastases are so named because the tumor cells “drop” from the primary site in the cerebellum to lower regions of the spinal cord, leading to the formation of secondary tumor nodules. Drop metastases are a significant concern in medulloblastoma as they indicate more advanced disease and are associated with a poorer prognosis [7]. However, in some rare cases, primary leptomeningeal medulloblastoma has been diagnosed in the absence of cerebellar involvement [9–13].

## 2. Case Presentation

### 2.1. History

A 34-year-old female with no significant past medical history presented for emergent evaluation in early December 2023 after experiencing acute severe bilateral lower extremity radiculopathy. She had no neurologic deficits on examination, so she was discharged from the emergency department without imaging. Over the course of the next 2 weeks, she experienced progressive numbness, spasms, and weakness, particularly in her right leg, eventually requiring a wheelchair for ambulation. These progressive deficits and new-onset urinary retention prompted her return to the emergency department, where she was found to have reduced strength of the right lower extremity on hip flexion, knee extension, dorsiflexion, and plantarflexion. Sensation was decreased throughout the entire right lower extremity. Additionally, her bicep, triceps, brachioradial, patellar, and ankle reflexes were decreased bilaterally.

**Figure 1:**
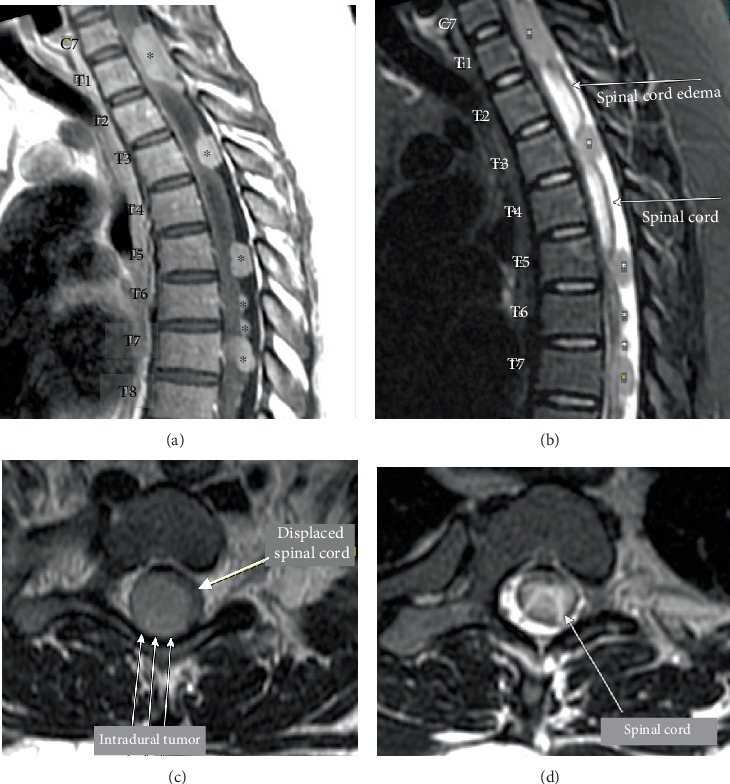
Preoperative images. Preoperative (a) sagittal T1 with contrast and (b) sagittal T2 demonstrating multiple compressive tumors (⁣^∗^) throughout the lower cervical and thoracic spine. Preoperative T2 axial MRI demonstrating an (c) intradural tumor with displaced spinal cord and (d) spinal cord edema.

Subsequent cervical, thoracic, and lumbar MRI demonstrated multiple moderately contrast-enhancing T2 isointense intradural, extramedullary lesions (Figure 1). These tumors were most prominent at C7 and T1, measuring approximately 3.3 cm eccentric to the right, with significant mass effect on the spinal cord. Additional lesions were observed at T3 and from T5 to T7 with a syrinx from C5 to T5. Brain MRI was unremarkable for any pathology. The patient was then emergently transferred to our facility where she underwent a C7–T5 laminectomy with resection of multiple tumors at C7, T2, and T4.

### 2.2. Operation

The C7 tumor was firm and adherent to the spinal cord. Bipolar cautery was used to remove the capsule, which demonstrated an atypical lymphoid infiltrate. Because the preliminary diagnosis suggested a pathologic process that would be responsive to medical therapy, the decision was made to debulk the lesions with an ultrasonic aspirator. Gross total resection was not achieved because the tumors could not be safely separated from the spinal cord.

### 2.3. Pathology

**Figure 2:**
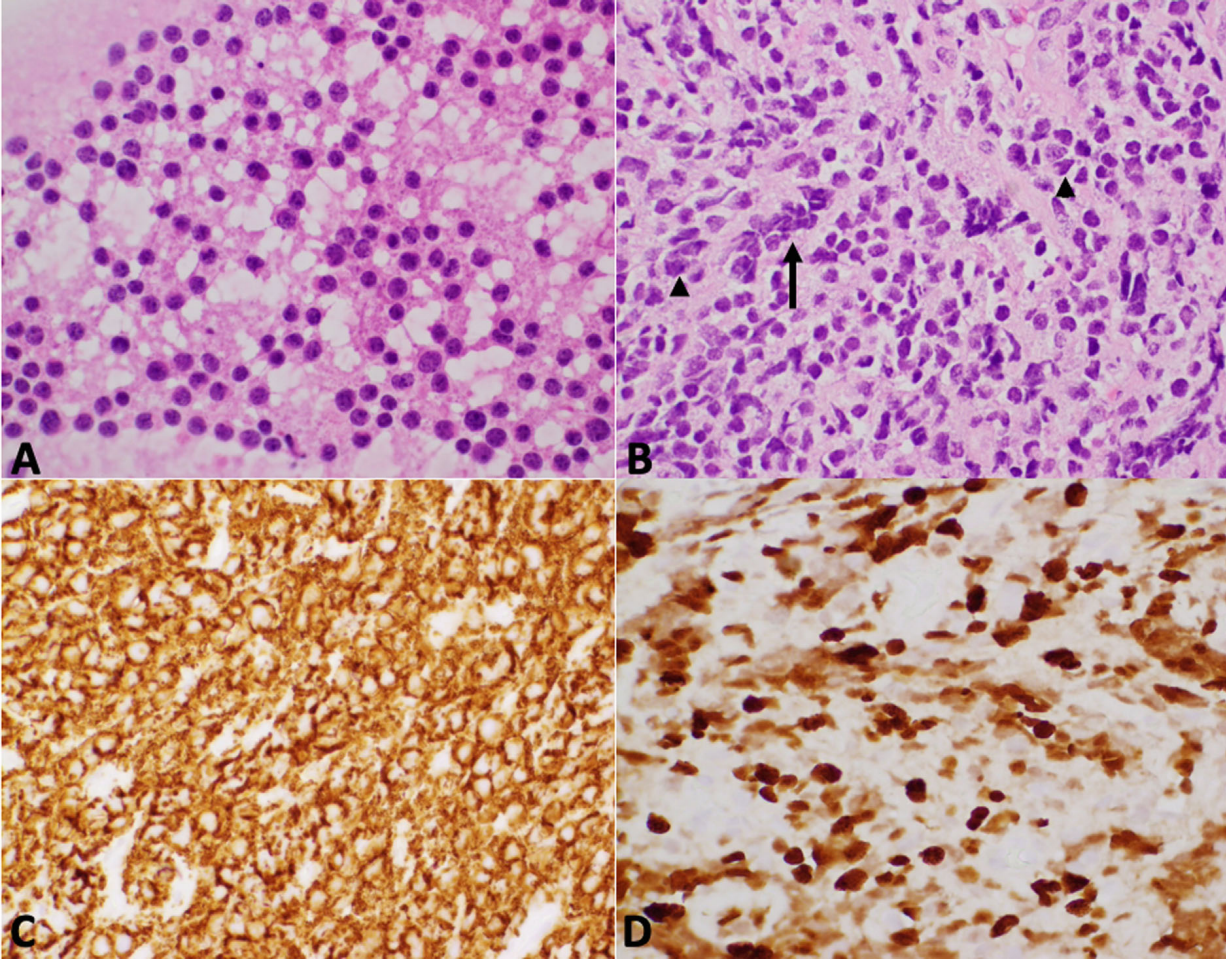
Specimen pathology. (A) Touch preparation of the tumor showing small to intermediate cells with minimal cytoplasm. (B) Paraffin section stained with hematoxylin and eosin (H&E) showing vaguely nested pattern (arrow) and focal nuclear molding (arrowheads). (C) Immunohistochemical study for CD56 showing strong, diffuse reactivity. (D) Ki67 (MIB1) showing many labeled tumor cell nuclei.

Biopsy material showed an atypical infiltrate consisting of small to intermediate sized cells with round to irregular nuclear contours and minimal cytoplasm in a vaguely nested pattern of growth (Figure 2). The tumor was positive for CD56, synaptophysin, and glial fibrillary acidic protein. The tumor showed no evidence of lymphoproliferative neoplastic behavior. Next-generation sequencing revealed a *PTCH1* mutation but no mutations of *TP53*, *H3K27M* (*K28*), *IDH*, or amplifications of *MYC* or *MYCN*. Additional mutations included POLE, LYST, KMT2D, and TERT. Further studies performed at St. Jude Children's Research Hospital confirmed activation of the SHH pathway and, by DNA methylation profiling identified a “methylation class medulloblastoma, group SHH, Subgroup 4” with high confidence (calibrated score = 0.99). Despite the lack of a posterior fossa mass, these results were compatible with a diagnosis of “medulloblastoma, SHH-activated, and *TP53-*wildtype.”

### 2.4. Postoperative Course

**Figure 3:**
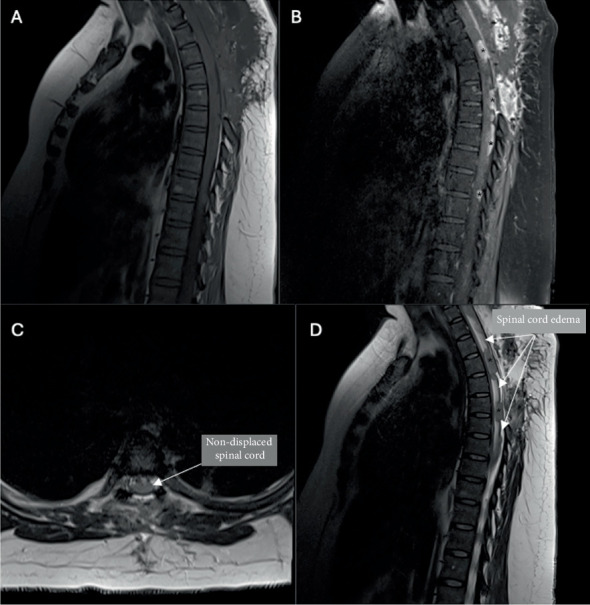
Postoperative images. Postoperative T1 sagittal MRI (A) without contrast and (B) with contrast demonstrating residual tumor with significantly less volume (⁣^∗^) and spinal cord compression. Postoperative (C) T2 axial MRI without displaced cord and (D) T2 sagittal MRI with continued spinal cord edema.

Consistent with preoperative findings, postoperative MRI of the brain demonstrated normal morphology. Cervical MRI showed C7 enhancement within the bilateral neural foraminal regions with a focal area of posterior enhancement. Marked spinal cord edema was seen from C4 to C7, though cervical alignment, osseous structures, and intervertebral discs were normal. Posterior decompression of the thoracic spine was seen to the level of T6 (Figure 3). The thoracic spine showed diffuse enhancing lesions, the most prominent of which were located at the posterior aspect of T1, T3, T5–T6, and T7. A dominant L2 lesion was seen on lumbar MRI. Thoracic and lumbar spine alignment was normal.

While in the ICU, the patient became unstable and developed a fever, respiratory distress, and worsening neurologic function, with her lower extremities progressing to complete paralysis. After this decline, the patient underwent 10 days of C5–T9 intensity modulated radiation therapy totaling 3000 Gy. Some improvements in strength, sensation, and range of motion were reported. She was discharged to an inpatient rehabilitation center at an outside institution near her home. The patient was reported to be receiving oncology care 3 months after surgery.

## 3. Discussion

While dissemination of tumor cells can occur at any time during medulloblastoma disease progression, this process is most often secondary to the development of a primary cerebellar lesion [14–16]. This report ultimately contributes to the limited literature on primary leptomeningeal medulloblastoma without a cerebellar mass in adults. To our knowledge, only five other cases of this nature have been reported (Table 1) [9–13]. These cases, like our own, present with nonspecific symptoms such as pain, neurological deficits, and progressive loss of function. However, our report describes the first case of primary leptomeningeal medulloblastoma localized purely to the spine with no cranial-related symptoms. Furthermore, our patient presented with multiple tumors across the entire spine, whereas other reports in the literature are localized to a single lesion. The rarity of this disease and similar presentation with other spinal pathologies underscore the importance of maintaining a broad differential diagnosis when evaluating adults with spinal lesions and neurological symptoms.

One key aspect of this case that differs from other reports is the widespread tumor burden throughout the spine. While other reports of primary leptomeningeal medulloblastoma were localized to a single spinal region, this is the first report of primary leptomeningeal medulloblastoma involving the entire spine, possibly in the setting of rapid tumor progression. Specifically, our patient experienced a rapid decline in neurologic functioning over the course of 1 month from the initial onset of symptoms. Genetic analysis of our patient's tumor revealed mutations consistent with the SHH-activated TP53-wildtype subtype of medulloblastoma. This subtype conveys an intermediate prognosis with a 5-year overall survival reported as approximately 75% [17–20]. Our patient had additional mutations at POLE, LYST, KMT2D, and TERT, all of which have been shown to significantly reduce overall survival in medulloblastoma [17, 19, 21–24]. The hypermutated tumor profile seen in our patient may have been driven by the POLE mutation, which encodes a proofreading polymerase known to drive hypermutation in medulloblastoma [25, 26]. Thus, the constellation of these additional mutations may have exacerbated tumor growth, contributing to the rapid spread across the patient's entire spine.

Given the unique nature of this case, future research should be conducted to better identify, classify, and treat primary leptomeningeal medulloblastoma. For one, there is a need for comprehensive epidemiological studies to ascertain the true incidence and prevalence of this rare tumor. An enhanced understanding of genetic factors and how these mutations contribute to aggressive tumor behavior and widespread spinal involvement is crucial. This could be accomplished with further genetic and molecular profiling studies specific to primary leptomeningeal medulloblastoma. Studies may additionally work to discover biomarkers that aid in earlier detection and prognostication of primary leptomeningeal medulloblastoma, potentially guiding personalized treatment strategies. Prospective trials may look to utilize more frequent or novel imaging modalities and create diagnostic algorithms tailored for early detection in adults presenting with nonspecific neurological symptoms.

In sum, advancing knowledge in these areas holds promise for improving outcomes for patients with primary leptomeningeal medulloblastoma. Additional cases of this tumor type should be reported to drive research and clinical practice forward.

## Figures and Tables

**Table 1:** Review of reported primary leptomeningeal medulloblastoma in adults as of July 2024.

**Age (years)/gender**	**Symptoms and neurologic signs**	**Lesion location**	**Treatment**	**Outcome**
21/M [10]	Headache, diplopia, tinnitus, dysmetria, positive left-sided Babinski's sign, elevated intracranial pressure (250 mm H_2_O), and triventricular hydrocephalus	Thoracic spine	Emergency posterior fossa craniotomy was performed where biopsy material revealed medulloblastoma. Radiation therapy was initiated 18 days after craniotomy for 1 day and was stopped due to loss of consciousness and spontaneous breathing	Patient died 6 months after surgery.
18/F [12]	Headache, blurry vision, right leg weakness, nausea, vomiting, bilateral CN VI palsy, and Grade III papilloedema	T6–T7	Laminectomy with excisional biopsy	Patient is alive and demonstrated a remarkable improvement in vision and headaches. CN VI palsy remains.
34/M [9]	Diplopia	L3–L4	Laminectomy with gross total tumor resection and radiotherapy	Patient died 6 weeks after surgery.
54/F [11]	Quadriplegia, hypotonia in all limbs, and bladder and bowel dysfunction	C2–C5	Laminectomy and radiotherapy	Breathing and limb strength improved, but constipation and urinary retention remain.
30/M [13]	Persistent nausea, vomiting, and neck pain 3 months before admission. Patient was unresponsive on admission secondary to obstructive hydrocephalus	C3–C4	No treatment for the lesion was performed; however, the patient received a ventriculostomy on admission for obstructive hydrocephalus	Patient died during admission.

## Data Availability

Research data are not shared.

## Nomenclature

ICU: intensive care unit
MRI: magnetic resonance imaging
SHH: sonic hedgehog
